# Port‐site incisional hernia from an 8‐mm robotic trocar following robot‐assisted radical cystectomy: Report of a rare case

**DOI:** 10.1002/iju5.12155

**Published:** 2020-03-21

**Authors:** Shoko Uketa, Yousuke Shimizu, Kosuke Ogawa, Noriaki Utsunomiya, Sojun Kanamaru

**Affiliations:** ^1^ Department of Urology Kobe City Nishi‐Kobe Medical Center Kobe Japan

**Keywords:** 8‐mm trocar, complication, cystectomy, port‐site incisional hernia, robotic surgery

## Abstract

**Introduction:**

Port‐site incisional hernia is a rare but well‐known complication following a laparoscopic procedure and it may cause severe adverse outcomes, such as intestinal necrosis. Here, we report a rare case of hernia that occurred from an 8‐mm trocar after robot‐assisted radical cystectomy.

**Case presentation:**

An 80‐year‐old woman was diagnosed with cT2bN1M0 bladder cancer. She underwent robot‐assisted radical cystectomy. Nine days after surgery, she complained of severe abdominal pain. Computed tomography showed herniation of small intestine. Emergency explorative laparotomy revealed herniation of small intestine from an 8‐mm trocar site. A section of the small bowel was necrotic and was resected.

**Conclusion:**

It is debatable whether we should routinely close the fascia of an 8‐mm trocar site. The patient was an elderly woman with multiple major abdominal surgery histories and hernia risk factors. For these patients, fascial closure of the 8‐mm trocar site may be indicated.

**Abbreviations & Acronyms**
CT: computed tomography
MRI: magnetic resonance imaging
PIH: port‐site incisional hernia
POD: postoperative day
RALAR: robot‐assisted low anterior resection
RALH: robot‐assisted laparoscopic hysterectomy
RALP: robot‐assisted laparoscopic prostatectomy
RARC: robot‐assisted radical cystectomy
RC: robotic cholecystectomy

**Keynote message**

We experienced a rare case of PIH from an 8‐mm trocar site after RARC. It is debatable whether we should routinely close the fascia of an 8‐mm trocar site. Further study is necessary to elucidate the indication for fascial closure of trocar sites after robotic surgery.

## Introduction

Today, robot‐assisted laparoscopic surgery is gaining widespread use in many surgical fields, as well as in the urology field. PIH is a rare complication that can occur during laparoscopic and robot‐assisted laparoscopic procedures, although PIH from an 8‐mm trocar is even rarer. PIH may lead to bowel obstruction and emergency surgery. There are not enough data about PIH to establish its prevalence, and we can find few cases of PIH from an 8‐mm trocar site following robot‐assisted surgery. Here, we describe our patients with PIH from an 8‐mm trocar and review the reports of such cases.

## Case presentation

**Fig. 1:**
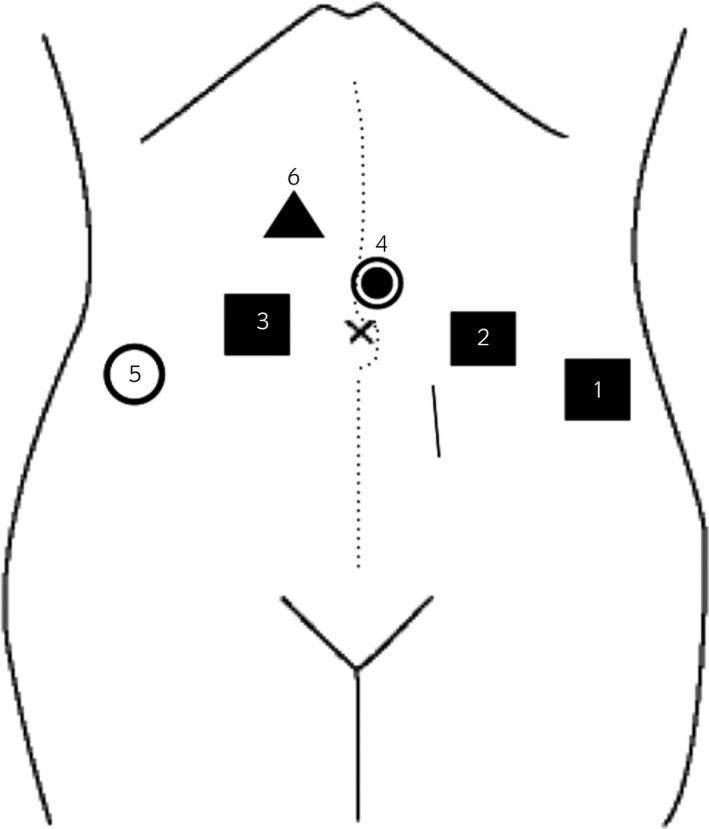
Trocar placement for RARC. Squares (1–3) represent 8‐mm robot arm ports. Square 2 was the location of hernia. Circles (4,5) represent 12‐mm port and triangle (6) represents 5‐mm assistant port. Dotted line represents previous surgical scar. Solid line represents first laparoscopic camera port.

**Fig. 2:**
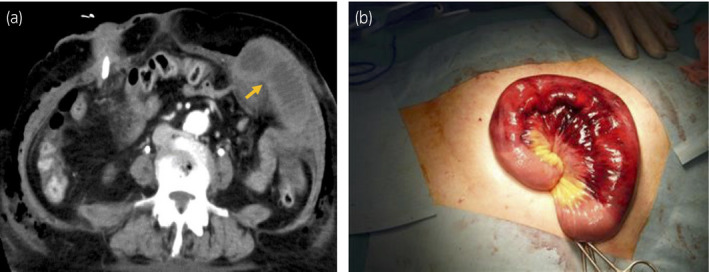
(a) Computed tomography showed the small intestinal herniation from the left port site. The arrow shows hernial orifice. (b) Surgeons showed engorgement of the small intestine and discoloration of bowel loops.

An 80‐year‐old Japanese woman (height 155.2 cm, weight 58.5 kg, body mass index 24.3 kg/m^2^) was admitted to our hospital because of macroscopic hematuria for 1 month. Cystoscopy revealed a nodular tumor filling the left wall of the bladder; CT and MRI showed cT2bN1M0 bladder cancer and left hydronephrosis. She underwent transurethral resection of the bladder cancer and was diagnosed with high‐grade pT2 < urothelial carcinoma. She underwent left percutaneous nephrostomy catheter placement and received three courses of neoadjuvant chemotherapy with gemcitabine and cisplatin. Thereafter, she underwent RARC with an extracorporeal ileal conduit. She previously underwent open surgeries for an ectopic pregnancy and traumatic splenic injury; her body had surgical scars extending from under the xiphoid process to the upper rim of the pubic bone. Because intestinal or abdominal adhesions were assumed to be present, the camera port was first placed in the lower left abdomen and laparoscopic lysis of abdominals was performed. Then, we closed the first camera port and relocated the port for the da‐Vinci camera above the navel. Other ports were placed as described in Figure 1. The total operative and console time were 836 min and 557 min, respectively. Estimate blood loss was 313 mL. Insufflation pressure was 10 mmHg. We closed the fascia of the AirSeal^®^ (SurgiQuest, Inc, Milford, CT, USA) access port and camera port. The early postoperative period was uneventful. Nine days after surgery, she complained of severe abdominal pain and nausea. Clinical examination revealed a distended abdomen. Abdominal CT revealed herniation of the small intestinal from the 8‐mm trocar site (Fig. 2a). An emergency explorative laparotomy revealed that the small intestine was partially prolapsed from the 8‐mm trocar and strangulated, causing engorgement of the small intestine and discoloration of bowel loops (Fig. 2b). The strangulation was released, but there was no improvement in blood flow in some sections of the small bowel, so intestinal resection and reconstruction was performed. She was discharged 35 days after surgery, and her clinical course was uneventful through follow‐up.

## Discussion

PIH is a rare but well‐known complication of laparoscopic surgery, and it may have cause severe adverse outcomes such as intestinal necrosis.^1^ Montz *et al*.^2^ reported that the occurrence of PIH following laparoscopic surgery has been calculated as 21 per 100 000 laparoscopic surgeries, and most PIH cases were associated with trocars >10 mm in diameter, while only 2.7% occurred with the use of trocars <8 mm in diameter. The recent literature on PIH in robot‐assisted urologic surgery reported an incidence of 0.66% with a predilection for periumbilical 12‐mm trocars.^3^ To the best of our knowledge, only eight patients with PIH from an 8‐mm robotic trocar site have been reported (Table 1).^4^, ^5^, ^6^, ^7^, ^8^, ^9^, ^10^ In all of these patients, the fascia of the trocar sites was left open. In most cases, PIH occurred within 1 week after surgery, but one patient experienced PIH more than 2 years after surgery,^9^ and another developed asymptomatic PIH.^6^ All patients required surgical intervention, and seven patients, including our case, required bowel resection.

**Table 1:** Summary of reports of nine patients with PIH from an 8‐mm robotic port site

No.	Author	Sex	Age	Procedure	Trocar obturator	Fascial closure	Occurrence time	Bowel resection
1	Seamon *et al.* ^4^	Female	67	RALH	Bladeless	No	POD 4	Yes
2	Spaliviero *et al.* ^5^	Male	Not mentioned	RALP	Not mentioned	No	POD 14	Yes
3	Fuller *et al.* ^6^	Male	Not mentioned	RALP	Not mentioned	No	Not mentioned	Yes
4	Fuller *et al.* ^6^	Male	Not mentioned	RALP	Not mentioned	No	Not mentioned	No
5	Tsu *et al.* ^7^	Male	75	RALP	Sharp	No	POD 4	Yes
6	Kilic *et al.* ^8^	Female	53	RALH	Not mentioned	No	POD 3	Yes
7	Lim *et al.* ^9^	Male	70	RALAR	Bladeless	No	After 32 months	No
8	Cho *et al.* ^10^	Female	37	RC	Not mentioned	No	POD 3	Yes
9	Our case	Female	80	RARC	Sharp	No	POD 9	Yes

PIH is considered to result from patient factors and technical factors.^3^ Patient factors are age, gender, obesity, previous abdominal surgery,^11^ postoperative factors resulting in increased intra‐abdominal pressure, such as constipation or cough,^6^ and factors affecting wound healing, such as diabetes mellitus, chemotherapy, infection, smoking, and malnutrition.^12^, ^13^ Technical factors are operative time, trocar shape, movement of robot arms, and port position. The tip of the trocar‐obturator is designed to be very sharp to easily pass through the fascia; therefore, this leads to a bigger incision in the fascia. Robot arms have a range of motion wider than that of the usual laparoscopic hand motion, which causes the incision to spread. Robotic arms are inserted more laterally than the usual laparoscopic trocar placement. We place each trocar 8 cm away from other trocars to prevent robotic arm collision.^8^ This ultimately pushes the robotic trocar to a location where the abdominal fascia becomes weaker. We considered No. 2 trocar site (Fig. 1) was placed near to the midline than others; furthermore, fascial closure of the first camera port which was near to No. 2 caused fascial tear and weakening. We believe that our patient had many risk factors leading to PIH, including older age, female gender, previous abdominal surgery, prolonged operative time, robotic surgery, sharp tip of trocar‐obturator, and lateral port position. From the lessons learned in this case, we now close the fascia of 8‐mm trocar sites with the Endo Close^TM^ trocar site closure device (Medtronic, Minneapolis, MN, USA) under direct vision laparoscopically, and PIH has not occurred in any patient. However, it is debatable whether we should routinely close the fascia of an 8‐mm trocar after robotic surgery. Mahmoud *et al*.^14^ suggested that the trocar should be placed away from the midline of the abdomen at an angle of 40–60° to the abdominal wall for avoiding hernia development. Because the occurrence rate of robotic 8‐mm trocar hernias is very low, more case reports are necessary to determine the risk factors for an 8‐mm trocar hernia and which patients need to have these trocar sites closed.

## Conclusion

We experienced a rare case of PIH from an 8‐mm trocar after RARC. PIH from an 8‐mm trocar is very rare but may lead to serious adverse outcomes, such as intestinal necrosis. Further study is necessary to elucidate the indication for fascial closure of an 8‐mm trocar site.

## Conflict of interest

The authors declare no conflict of interest.
